# Automatic detection of mild cognitive impairment based on deep learning and radiomics of MR imaging

**DOI:** 10.3389/fmed.2024.1305565

**Published:** 2024-01-12

**Authors:** Mingguang Yang, Shan Meng, Faqi Wu, Feng Shi, Yuwei Xia, Junbang Feng, Jinrui Zhang, Chuanming Li

**Affiliations:** ^1^Medical Imaging Department, Chongqing Emergency Medical Center, Chongqing University Central Hospital, Chongqing, China; ^2^Department of Radiology, Chongqing Western Hospital, Chongqing, China; ^3^Department of Medical Service, Yanzhuang Central Hospital of Gangcheng District, Jinan, China; ^4^Department of Research and Development, Shanghai United Imaging Intelligence, Co., Ltd., Shanghai, China

**Keywords:** MCI, AD, deep learning, radiomics, MRI

## Abstract

**Purpose:**

Early and rapid diagnosis of mild cognitive impairment (MCI) has important clinical value in improving the prognosis of Alzheimer’s disease (AD). The hippocampus and parahippocampal gyrus play crucial roles in the occurrence of cognitive function decline. In this study, deep learning and radiomics techniques were used to automatically detect MCI from healthy controls (HCs).

**Method:**

This study included 115 MCI patients and 133 normal individuals with 3D-T1 weighted MR structural images from the ADNI database. The identification and segmentation of the hippocampus and parahippocampal gyrus were automatically performed with a VB-net, and radiomics features were extracted. Relief, Minimum Redundancy Maximum Correlation, Recursive Feature Elimination and the minimum absolute shrinkage and selection operator (LASSO) were used to reduce the dimensionality and select the optimal features. Five independent machine learning classifiers including Support Vector Machine (SVM), Random forest (RF), Logistic Regression (LR), Bagging Decision Tree (BDT), and Gaussian Process (GP) were trained on the training set, and validated on the testing set to detect the MCI. The Delong test was used to assess the performance of different models.

**Result:**

Our VB-net could automatically identify and segment the bilateral hippocampus and parahippocampal gyrus. After four steps of feature dimensionality reduction, the GP models based on combined features (11 features from the hippocampus, and 4 features from the parahippocampal gyrus) showed the best performance for the MCI and normal control subject discrimination. The AUC of the training set and test set were 0.954 (95% CI: 0.929–0.979) and 0.866 (95% CI: 0.757–0.976), respectively. Decision curve analysis showed that the clinical benefit of the line graph model was high.

**Conclusion:**

The GP classifier based on 15 radiomics features of bilateral hippocampal and parahippocampal gyrus could detect MCI from normal controls with high accuracy based on conventional MR images. Our fully automatic model could rapidly process the MRI data and give results in 1 minute, which provided important clinical value in assisted diagnosis.

## Introduction

Alzheimer’s disease (AD) is an irreversible chronic neurodegenerative brain disease that poses a serious threat to human health. The main clinical manifestations include memory impairment, aphasia, loss of use and recognition, impairment of visual and spatial skills, executive dysfunction, and personality and behavioral changes (1). The occurrence and development of AD is a continuous process, and mild cognitive impairment (MCI) is considered as the preclinical stage of AD (2, 3). Early diagnosis and timely treatment of MCI can delay the disease progression and have important clinical value to improve the prognosis (2–4).

At present, the diagnosis of MCI still relies on subjective clinical symptoms. Objective examination methods are urgently needed in clinical practice. FDG-PET and Amyloid-PET are expensive and need to be exposed to radiation, which limits their usefulness (5). As a medical imaging technique, MRI has the advantages of non-invasive, non-radiation exposure, and high-resolution capabilities, making it widely used in the diagnosis and staging of neurological diseases. As an important part of emotion regulation, the hippocampus and parahippocampal gyrus play key roles in cognitive function, especially emotional memory (6, 7). Recent studies have reported that the morphology and network connectivity changes of the hippocampus and parahippocampal gyrus were important indicators of MCI and AD (8–11). However, these studies mainly focused on macroscopic markers, but overlooked the small structural indicators. Lambin et al. proposed radiomics in 2012, which could help diagnose and differentiate diseases by quantifying the subtle information in medical images that were difficult to assess with the naked eye (12). Radiomics has shown important application value in many neurology diseases, such as PD, AD, epilepsy, and brain tumors (13–17). Previously, a radiomics study by Zhang et al. suggested that 3D textures of the hippocampus and entorhinal cortex might be a diagnostic biomarker for AD (18). Luk et al. used the hippocampus texture features of MRI to predict the conversion of mild cognitive impairment to AD with an accuracy of 76.2% (19). However, most of these literatures used manual methods to segment the brain region and extract relevant parameters, which were time-consuming, taking approximately 4 h to process a patient. These shortcomings limited their clinical use greatly. In this study, we developed a CNN-based artificial intelligence model for the automatic segmentation and radiomics features extraction of bilateral hippocampus and parahippocampal gyrus, and established diagnostic models to help distinguish between MCI and HC in a short time.

## Materials and methods

### Patient information

All data in this study were collected from the Alzheimer’s disease Neuroimaging Initiative (ADNI) database.^1^ This study was approved by the ethics standards committee of our institution. Totally 248 subjects with 3D-T1 weighted MR structural images, including 115 MCI patients and 133 normal individuals were included. According to the ADNI protocol, the diagnostic criteria for MCI should meet: (a) Cognitive problems reported by participants or those around them; (b) The patient showed impairment in the subtest logical memory-II on the Wechsler Memory Scale R; (c) Mini-Mental State Examination (MMSE) score ≥ 24. Clinical information was obtained for all participants, including age, sex, education, and MMSE score (Table 1).

**Table 1:** The demographic data of MCI and HC groups.

	MCI	HC	*t*/Z/F	*p*-value
Sample size	115	133	-	-
Gender, female (%)	50 (43.48)	80 (60.15)	6.873	0.009
Age (years)	72.43±7.88	69.13±7.25	−3.441	0.001
Education (years)	16.55±2.36	16.68±2.12	0.480	0.632
MMSE [*M* (*Q*1, *Q*3)]	27.75 (27, 29)	29.03 (29, 30)	−5.314	<0.001

MMSE, Mini-Mental State Examination.

### Image acquisition and preprocessing

For all participants, 3D-T1-MPRAGE or equivalent protocol with slimly different resolutions was used. ADNI website offered all of the detailed imaging parameters.^2^ For scanner 1 (Siemens Medical Solutions, 3.0 T), the scanning parameters were listed below: repetition time (TR) = 2300.0, echo time (TE) = 3.0, matrix = 240 × 256 × 176. For scanner 2 (General Electric Healthcare, 3.0 T), the scanning parameters were: TR = 7.7–7.0, TE = 3.1–2.8, matrix = 256 × 256 × 196. For scanner 3 (Philips Medical Systems, 3.0 T), the MR imaging data were acquired with the following parameters: TR = 6.8, TE = 3.1, matrix = 256 × 256 × 170. The layer thickness of the three different scanners was 1.0 or 1.2 mm, and the layer spacing was 0.

### The hippocampus and parahippocampal gyrus segmentation

The hippocampus and parahippocampal gyrus segmentation module was implemented using a deep learning algorithm based on a 3D VB-NET network (20). The data preprocessing module performed a series of operations, including rotation, resampling, resizing, skull stripping, image non-uniform correction, histogram matching, and gray-scale normalization on the MRI images used for training and testing. All images were standardized to the size of 256*256*256*1 mm^3^ in the standard Cartesian LPI coordinate system, and the gray-scale range was within the interval (−1, 1). The model was constructed based on 1,800 subjects and evaluation showed an averaged 0.92 Dice overlap with ground truth. The segmentation process took less than 1 minute for each patient.

### Radiomics features extraction

Totally 2,264 radiomics features were automatically extracted from the bilateral hippocampus or parahippocampal gyrus of each patient. The radiomics features included four categories of first-order features, shape features, texture features, and wavelet-based features (21). The first-order statistics and shape features could reflect the shape and size of the brain region. Texture features included Gray Level Co-occurrence Matrix (GLCM) features, Gray Level Run Length Matrix (GLRLM) features, Gray Level Size Zone Matrix (GLSZM) features, Neighboring Gray Tone Difference Matrix (NGTDM) features, and Gray Level Dependence Matrix (GLDM) features. The high-level features were obtained through 24 filters (including Box Mean, additive Gaussian Noise, binomial blur, curvature flow, Box-Sigma, normalization, Laplace Sharpening, discrete Gaussian, mean, speck noise, recursive Gaussian, Shot Noise and LoG with sigma values of 0.5, 1, 1.5 and 2), as well as wavelet transformations (LLL, LLH, LHL, LHH, HLL, HLH, HHL, and HHH).

### Radiomics features selection, models establishment and validation

**Figure 1:**
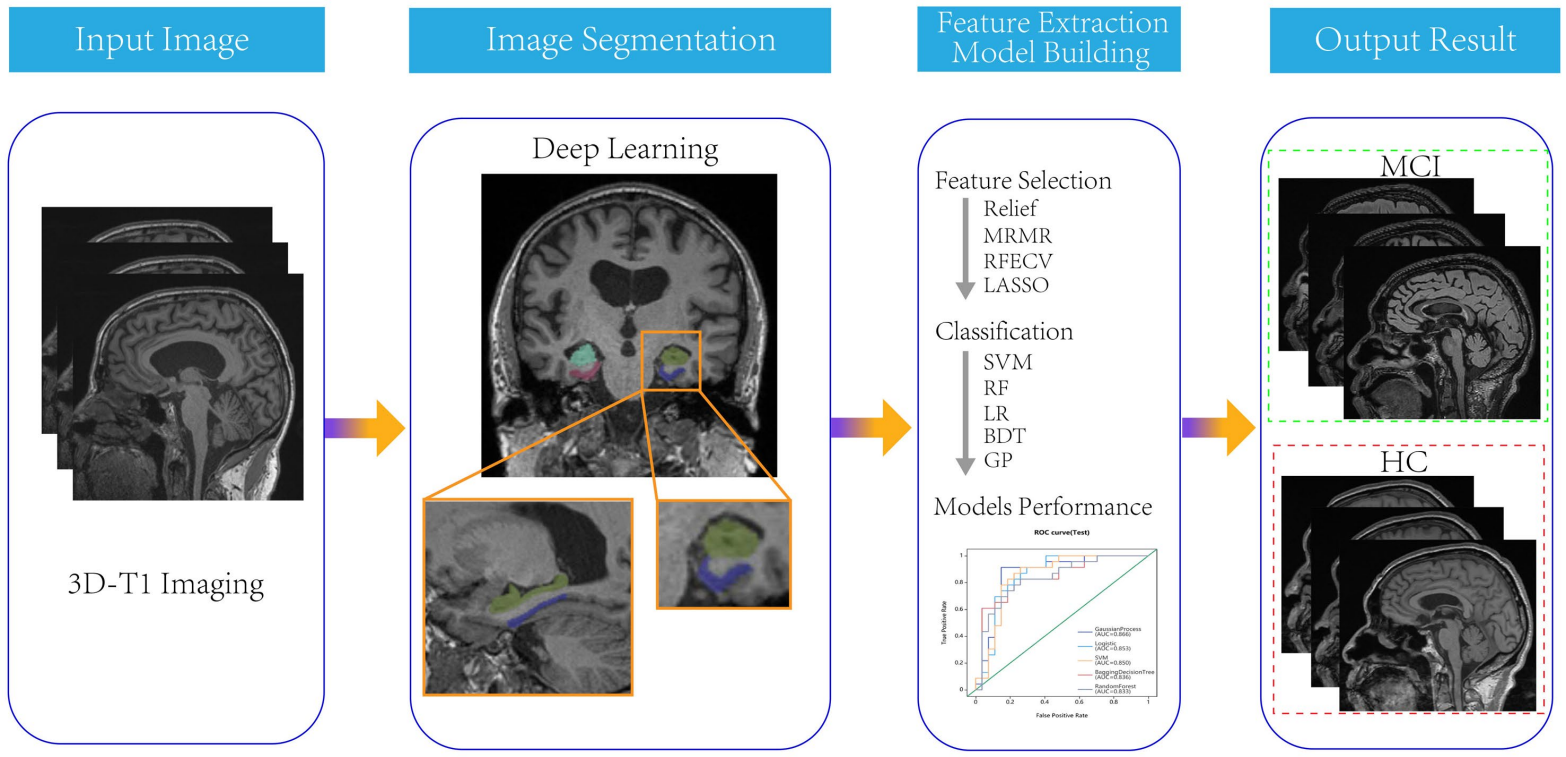
The flow chart of segmentation and models construction.

All patients were randomly divided into a training group and a testing group in an 8:2 ratio. Four feature selection methods, namely Relief, Minimum Redundancy Maximum Correlation, Recursive Feature Elimination, and LASSO were used to gradually select the optimal radiomics features. Then, five independent machine learning classifiers, including Support Vector Machine (SVM), Random forest (RF), Logistic Regression (LR), Bagging Decision Tree (BDT) and Gaussian Process (GP) algorithm were trained on the training set, and validated on the testing set in the form of 10 fold cross-validation. The flow chart of this study was shown in Figure 1.

### Statistics analysis

Statistical analysis was conducted using SPSS software (version 22.0, IBM). Quantitative data was tested for normality using the Kolmogorov–Smirnov method. Continuous variables with normal distribution were expressed as mean standard deviation and compared using independent sample *t*-tests. Continuous variables without normal distribution were expressed as median and compared using the Mann–Whitney U test. Classified variables were expressed in frequency (percentage) and compared using the chi-square test or Fisher’s exact test. The statistical significance was considered to be *p* < 0.05. The model performance was evaluated using the receiver operating characteristics (ROC) curve. The area under the curve (AUC), sensitivity, specificity, accuracy, as well as F1 score were calculated. The calibration curve was used to evaluate the calibration of the model, and DCA was used to evaluate the clinical applicability of the model.

## Results

Totally 115 MCI patients and 133 healthy controls were included in this study. There was no significant difference in educational level between the MCI and healthy control groups.

**Figure 2:**
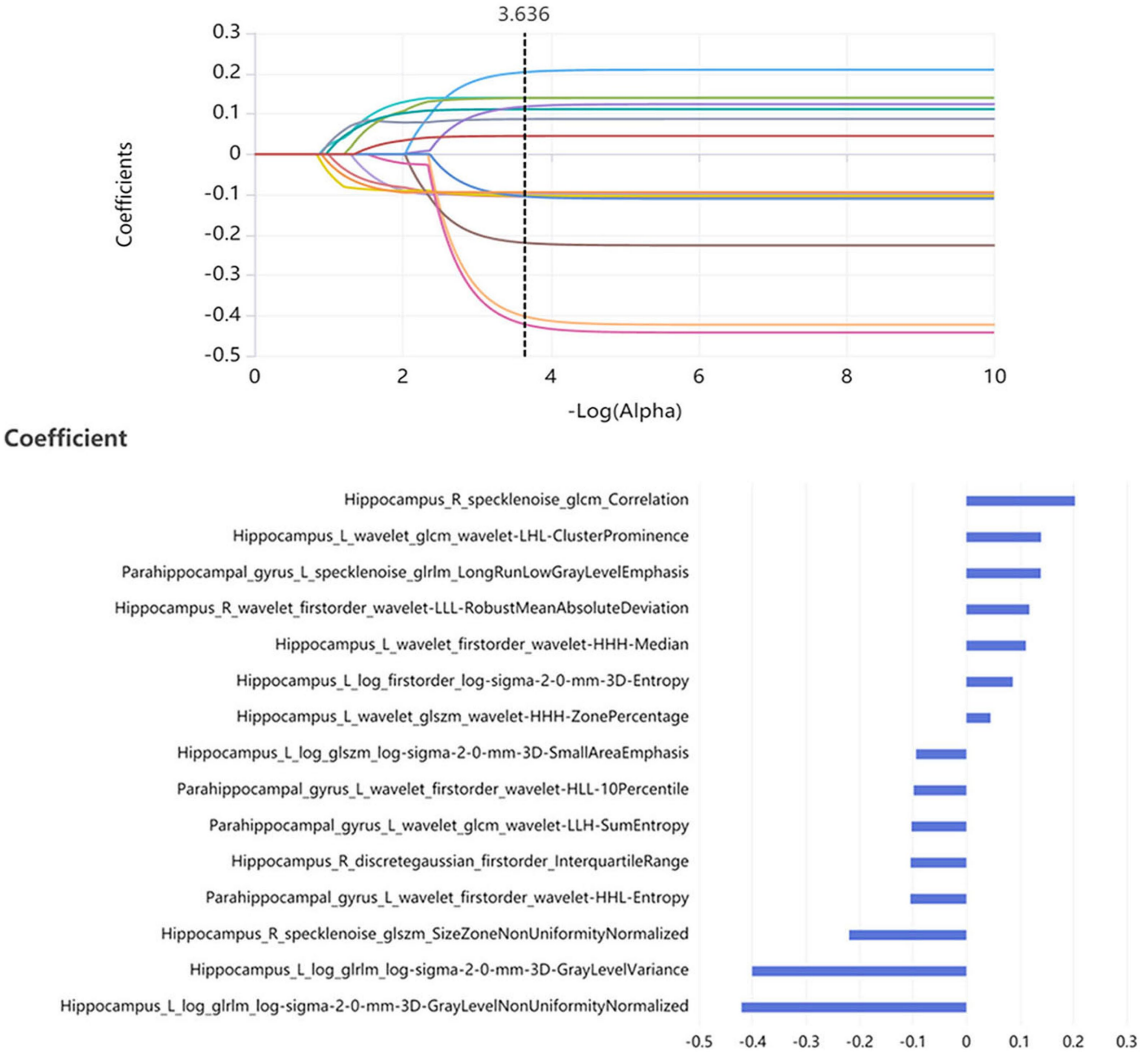
15 optimal radiomics features were obtained as the optimal combined features using the LASSO method.

In the training set, 200 features were selected from 4,528 radiomic features of bilateral hippocampus by features dimensionality reduction of the Relief, Minimum Redundancy Maximum Correlation and Recursive Feature Elimination methods. Then 13 optimal features were obtained using the LASSO method. According to the same method, 12 optimal features were obtained from the bilateral parahippocampal gyrus. 300 features were selected from radiomic features of both the bilateral hippocampus and parahippocampal gyrus by Relief, Minimum Redundancy Maximum Correlation and Recursive Feature Elimination methods, and then 15 optimal features were obtained as combined features using the LASSO method (Figure 2).

**Figure 3:**
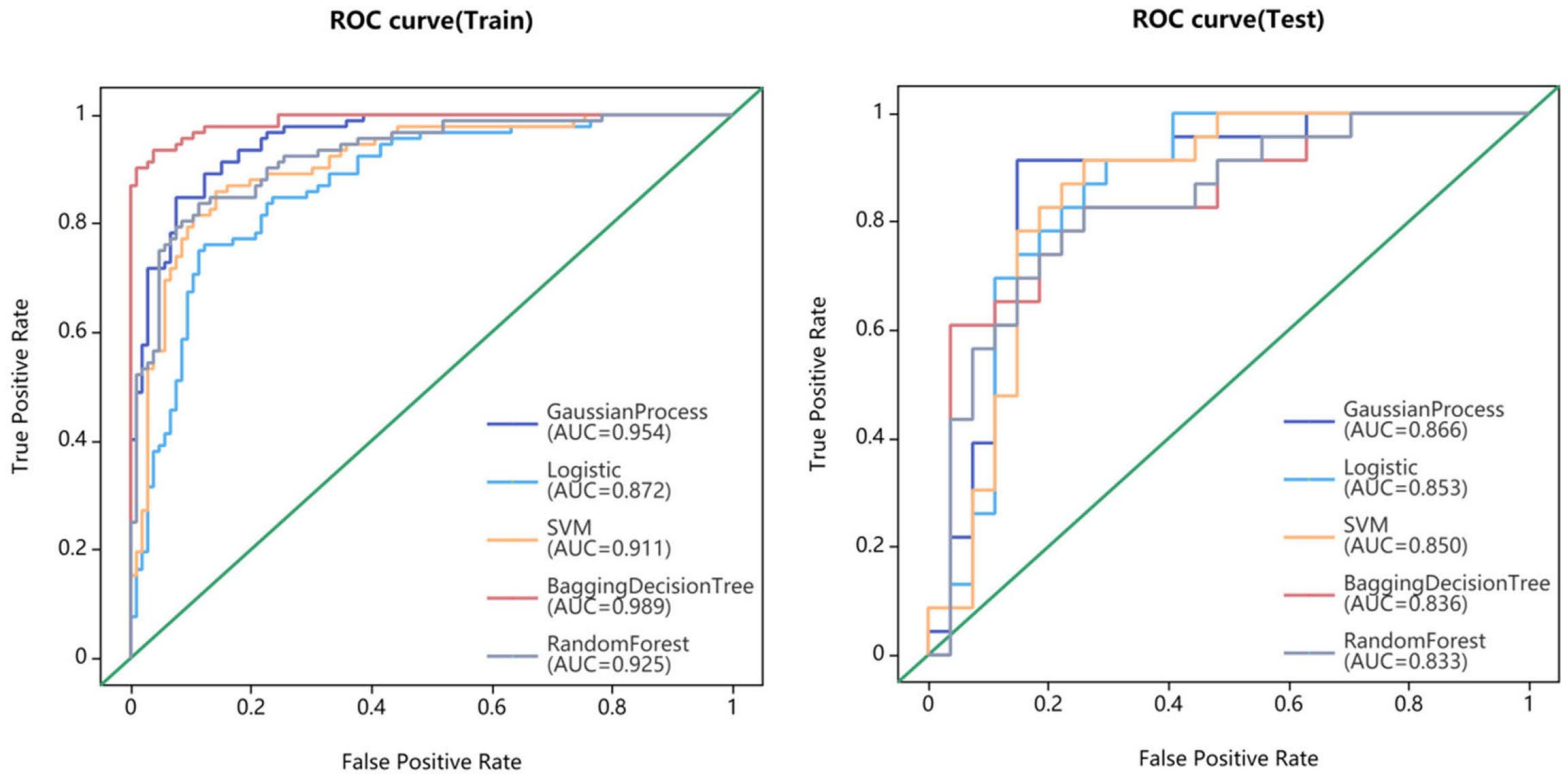
The models performance of GP, LR, SVM, BDT, and RF classifiers in the discrimination between MCI and normal controls.

**Figure 4:**
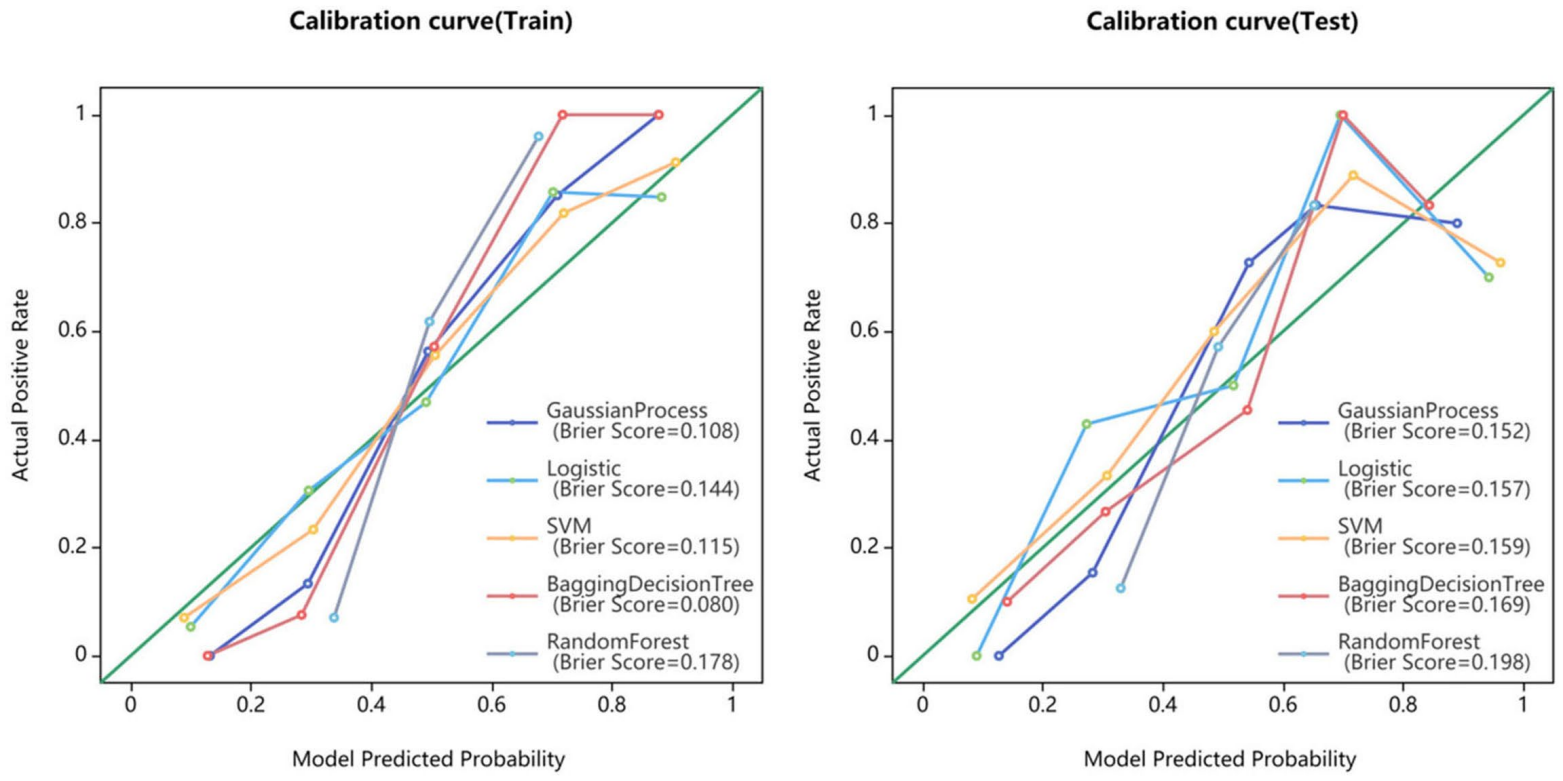
Calibration curves for GP, LR, SVM, BDT, and RF models.

**Figure 5:**
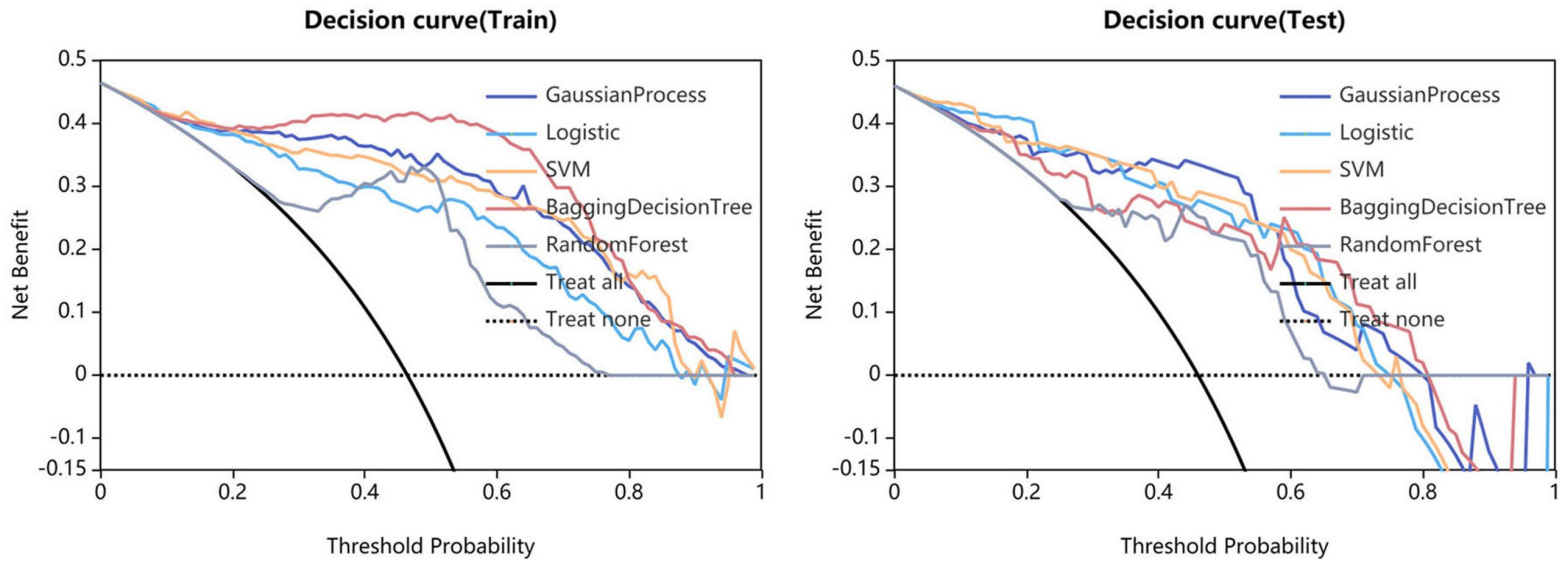
Clinical decision curves of GP, LR, SVM, BDT, and RF models.

Fifteen models were established based on the optimal features of the hippocampus, parahippocampal gyrus and combined features. ROC curves of GP, LR, SVM, BDT and RF models are shown in Figure 3. The DeLong test showed that GP models based on combined features (11 features from the hippocampus, and 4 features from parahippocampal gyrus) showed the best performance. The AUC of the training set and test set were 0.954 (95% CI: 0.929–0.979) and 0.866 (95% CI: 0.757–0.976), respectively. The sensitivity, specificity, and accuracy of the training set and test set were 0.848, 0.896, 0.874, and 0.870, 0.852, and 0.860, respectively (Table 2). The calibration curve showed a good agreement between the actual and predicted probabilities of the sample (Figure 4). Decision curve analysis showed that the GP model had the highest clinical net benefit (Figure 5).

**Table 2:** Performance of GP, BDT, SVM, RF, and LR models on training set and testing set.

		AUC		Sensitivity		Specificity		Accuracy	
	Models	Training set	Testing set	Training set	Testing set	Training set	Testing set	Training set	Testing set
Hippocampus	GP	0.912 (0.873–0.951)	0.839 (0.718–0.960)	0.761	0.783	0.849	0.889	0.808	0.840
	BDT	0.987 (0.974–0.999)	0.855 (0.749–0.961)	0.946	0.739	0.962	0.815	0.955	0.780
	SVM	0.888 (0.843–0.933)	0.810 (0.674–0.946)	0.793	0.739	0.821	0.815	0.808	0.780
	RF	0.873 (0.825–0.921)	0.815 (0.692–0.938)	0.522	0.565	0.925	0.926	0.737	0.760
	LR	0.863 (0.813–0.913)	0.794 (0.655–0.933)	0.750	0.826	0.821	0.778	0.788	0.800
Parahippocampal gyrus	GP	0.938 (0.907–0.968)	0.826 (0.709–0.943)	0.848	0.739	0.877	0.778	0.864	0.760
	BDT	0.968 (0.946–0.990)	0.736 (0.592–0.880)	0.913	0.739	0.877	0.630	0.894	0.680
	SVM	0.845 (0.790–0.900)	0.784 (0.650–0.918)	0.772	0.696	0.811	0.852	0.793	0.780
	RF	0.902 (0.861–0.944)	0.731 (0.586–0.876)	0.696	0.522	0.906	0.815	0.808	0.680
	LR	0.802 (0.741–0.862)	0.684 (0.528–0.840)	0.685	0.652	0.736	0.667	0.712	0.660
Combined features	GP	0.954 (0.929–0.979)	0.866 (0.757–0.976)	0.848	0.870	0.896	0.852	0.874	0.860
	BDT	0.989 (0.979–0.999)	0.836 (0.719–0.952)	0.913	0.739	0.962	0.815	0.939	0.780
	SVM	0.911 (0.870–0.952)	0.850 (0.737–0.964)	0.826	0.783	0.868	0.852	0.848	0.820
	RF	0.925 (0.889–0.961)	0.833 (0.717–0.948)	0.750	0.652	0.953	0.852	0.859	0.760
	LR	0.872 (0.823–0.922)	0.853 (0.740–0.967)	0.772	0.826	0.830	0.778	0.803	0.800

GP, Gaussian Process; BDT, Bagging Decision Tree; SVM, Support Vector Machine; RF, Random forest; LR, Logistic Regression.

## Discussion

With the aging of the population, the incidence of AD is increasing year by year. It had been proven that AD could be prevented, and the key lied in early detection of mild cognitive impairment (22–24). Therefore, developing a fast and accurate method to distinguish MCI and HC had become an important focus in clinical practice. Previous studies had reported that the morphological changes of hippocampal regions were closely related to the occurrence of MCI (25, 26). In this study, an automatic segmentation framework was established on 3D-T1 (MPRAGE) sequence images based on a 3D VB-NET deep learning model. The bilateral hippocampus and parahippocampal gyrus were automatically segmented and a large number of radiomic features were automatically extracted. We found that among the classifiers of GP, LR, SVM, BDT, and RF algorithms, the GP classifier had the highest classification performance, with an AUC of 0.954 in the training set and 0.866 in the test set. Our results showed that the application of artificial intelligence technology to analyze raw T1-weighted MRI images could accurately detect MCI from normal controls. This was a very objective method that did not rely on the patient’s personal medical history or the doctor’s clinical experience. Previously, Ferrarini et al. had used markers based on the shape of the hippocampus to distinguish between AD and MCI with an accuracy of 80% (27). A meta-analysis of the value of hippocampal volume in the diagnosis of MCI showed a sensitivity and specificity of 60 and 75%, respectively (28). Beheshti et al. used a voxel-based morphometric method to construct a structural connection network and a support vector machine (SVM) model to achieve a 70.38% accuracy for MCI and normal control discrimination (29). Feng et al. (30) developed a logistic regression machine learning model to identify MCI from normal control with an accuracy of 0.79 and 0.76 using radiomics features of the hippocampus. In these previous studies, the accuracies were low and many software such as VBM, SPM, Freesufer, 3DSlicer or Python were used to achieve manual brain region segmentation and features extraction, which greatly reduced the work efficiency. Compared to them, our method could achieve fully automated brain segmentation, feature extraction, and diagnostic modeling establishment. Our results were more accurate and the results could be obtained in several minutes. It had the characteristics of objectivity, high speed, low cost, and high accuracy, making it more suitable for clinical application and promotion.

Radiomics features contain much microstructure information that reflects the underlying early biomarkers of pathophysiology. In this study, the optimal model contained 15 radiomics features, including 11 features from the hippocampus and 4 features from the parahippocampal gyrus. The 11 features from the hippocampus included 4 first-order features, 3 GLSZM features, 2 GLRLM features, and 2 GLCM features. The four radiomic features of the parahippocampal gyrus included 2 first-order features, 1 GLCM features and 1 GLRLM features. The hippocampus is located between the thalamus and the medial temporal lobe of the brain and is part of the limbic system. It is mainly responsible for the storage, conversion, and orientation functions of short-term memory (31). The hippocampus is one of the earliest brain regions affected by Alzheimer’s disease. As the disease progresses, hippocampal damage gradually worsens, which can help determine the severity of the disease, monitor the progress of the disease, or evaluate the effectiveness of interventions such as medication, cognitive therapy, and healthy lifestyles (32). The parahippocampal gyrus is an important structure that assists the hippocampus in its function (33). The damage of them can cause abnormalities in emotion, cognition and behavior. The first-order features include mean absolute deviation, kurtosis, energy and minimum, which mainly reflect the basic statistical information of the image from various angles. It could measure the asymmetry and flatness of the morphological layout of the brain regions. Previously, Feng et al. had found hippocampal neuroanatomical abnormalities of size, shape, gray value distribution and spatial heterogeneity in MCI subjects (30). GLSZM, GLRLM, and GLCM belong to texture features. They are based on different grayscale matrices to evaluate the spatial distribution of pixel intensity. These features have been proven to be useful in studying neuropathological heterogeneity. When pathological changes occur in the internal structure of the brain, its smoothness, roughness, and heterogeneity can be reflected through GLSZM, GLRLM, and GLCM features. The texture features of hippocampal microstructure have been proven to reflect cognitive function in direct and indirect ways (9).

Our study had several limitations. Firstly, this was a retrospective cross-sectional study, which did not track the dynamic process of the radiomic features. A prospective longitudinal follow-up study in the future is needed. Secondly, the sample size of MCI patients was relatively small, and internal cross-validation was adopted; therefore, the generalization of the model needed to be further verified by a larger sample and external validation. Finally, in order to achieve rapid and fully automated diagnosis, this study only considered imaging information. Adding more clinical information and biological indicators could further increase accuracy.

## Conclusion

The GP classifier based on 15 radiomics features of bilateral hippocampal and parahippocampal gyrus could detect MCI based on conventional MR images with high accuracy. Our fully automatic model could rapidly process the MRI data and distinguish MCI and HCs in 1 minute. Our method was fast, simple, and accurate, which provided important clinical value in assisted diagnosis.

## Data availability statement

The datasets presented in this study can be found in online repositories. The names of the repository/repositories and accession number(s) can be found in the article/supplementary material.

## Ethics statement

The studies involving humans were approved by Alzheimer’s disease Neuroimaging Initiative. The studies were conducted in accordance with the local legislation and institutional requirements. The participants provided their written informed consent to participate in this study.

## Author contributions

MY: Conceptualization, Data curation, Formal analysis, Investigation, Methodology, Validation, Writing – original draft. SM: Conceptualization, Formal analysis, Investigation, Methodology, Project administration, Writing – original draft. FW: Conceptualization, Data curation, Formal analysis, Writing – original draft. FS: Data curation, Formal analysis, Methodology, Software, Supervision, Validation, Writing – original draft. YX: Methodology, Project administration, Software, Supervision, Validation, Writing – original draft. JF: Data curation, Formal analysis, Methodology, Supervision, Writing – original draft. JZ: Data curation, Investigation, Methodology, Writing – original draft. CL: Conceptualization, Funding acquisition, Investigation, Resources, Writing – original draft, Writing – review & editing.

## Footnotes

^1^ http://adni.loni.usc.edu

^2^ http://adni.loni.usc.edu/methods/documents/
